# A non-reflecting wave equation through directional wave-field suppression and its finite difference implementation

**DOI:** 10.1038/s41598-021-04064-3

**Published:** 2022-01-10

**Authors:** Teun Schaeken, Leo Hoogerbrugge, Eric Verschuur

**Affiliations:** Imaging Physics, Delft University of Technology, Lorentzweg 1, 2628 CJ Delft, The Netherlands

**Keywords:** Acoustics, Geophysics, Seismology

## Abstract

The acoustic wave equation describes wave propagation directly from basic physical laws, even in heterogeneous acoustic media. When numerically simulating waves with the wave equation, contrasts in the medium parameters automatically generate all scattering effects. For some applications - such as propagation analysis or certain wave-equation based imaging techniques - it is desirable to suppress these reflections, as we are only interested in the transmitted wave-field. To achieve this, a modification to the constitutive relations is proposed, yielding an extra term that suppresses waves with reference to a preferred direction. The scale-factor $\alpha$ of this extra term can either be interpreted as a penetration depth or as a typical decay time. This modified theory is implemented using a staggered-grid, time-domain finite difference scheme, where the acoustic Poynting-vector is used to estimate the local propagation direction of the wave-field. The method was successfully used to suppress reflections in media with bone tissue (medical ultrasound) and geophysical subsurface structures, while introducing only minor perturbations to the transmitted wave-field and a small increase in computation time.

## Introduction

Numerical simulations of propagating wave-fields in complex heterogeneous media play an essential role in the field of acoustical imaging. Through a combination of acoustic recordings and numerical wave equation simulations, images of physically inaccessible, vastly differently sized objects can be made. Despite their numerical nature, these simulations honour the physical laws of wave theory.

One of these acoustical imaging methods is known as reverse time migration (RTM)^1,2^. RTM is comprised of two modeling phases, a forward modeling of source data and a backward modeling of time-reversed receiver data. In its most basic form, RTM creates an image through a cross-correlation of forward modeled shot data and backward modeled, time-reversed, receiver data. In the past, this modeling phase was often performed using one-way propagators^3,4^. Nowadays, due to the increasing advances in computational processing and storage capabilities, finite difference modeling (FD) of the complete, two-way wave equation has become the preferred approach. A common problem in prestack-RTM is the formation of image artifacts due to the cross-correlation of unwanted reflections. These artifacts can be avoided directly, by suppressing these reflections within the simulation^5^, or indirectly, by means of angle-gathers^6,7^.

Recently, RTM, which has been developed for geophysical imaging, has also found its way to ultrasound applications like civil engineering^8,9^ and photo-acoustic imaging^10^. In medical applications, RTM has also shown to be successful in imaging breast-tissue using frequency domain finite difference modeling (FDFD)^11,12^. Other geophysical imaging methods, such as Full Waveform Inversion (FWI)^13^, have also been successfully applied to medical ultrasound imaging of high-contrast bone tissue, such as the human skull^14^. For these methods, distinguishing complex back-scattering from multiple arrivals by means of reflection suppression could serve as a useful tool within wave propagation analysis.

An obvious method to remove reflections is to smooth the inverse of the wave-speed (slowness) of the medium. This approach, however, is less effective for high-frequency components of the wave-field and comes at the expense of inaccurate wave propagation. Another popular method, matching the impedance inside the medium^15^, is only effective in suppressing reflections at small angles of incidence and does not preserve the original wave amplitudes. A final alternative is an approach where reflections are suppressed by virtue of predefined wave-field directions within carefully chosen regions^5^.

In this paper, we will build from the latter method towards a robust directional wave-field suppression theory. Using this theory and its implementation, waves are guided through the medium without reflections and without significant perturbations to the transmitted wave-field.

## Theory

Inside a lossless, heterogeneous, isotropic, acoustic medium the particle velocity and the acoustic pressure obey the equations of motion and the constitutive equation (stress-strain relation). Together, they can be written as a system of first-order hyperbolic partial differential equations (PDE’s):

1 $$\begin{aligned} \frac{\partial {\vec {v}}}{\partial {t}}&= -\frac{1}{\rho } \vec {\nabla }p, \end{aligned}$$

2 $$\begin{aligned} \frac{\partial {p}}{\partial {t}}&= - \rho c^2 \vec {\nabla }\cdot \vec {v}, \end{aligned}$$

where $v = v(\vec {r}, t)$ and $p = p(\vec {r}, t)$ denote the particle velocity and the acoustic pressure respectively, and $\rho = \rho (\vec {r})$ and $c = c(\vec {r})$ correspond to the density and compressional wave speed at each position $\vec {r}$ in the medium. In this paper, a modification of Eq. (2) is proposed to suppress wave-fields along a preferred direction:

3 $$\begin{aligned} \frac{\partial {\vec {v}}}{\partial {t}}&= -\frac{1}{\rho } \vec {\nabla }p,\end{aligned}$$

4 $$\begin{aligned} \frac{\partial {p}}{\partial {t}}&= - \rho c^2 \vec {\nabla }\cdot \vec {v} - \alpha \left( c p - \rho c^2 {\hat{S}} \cdot \vec {v} \right) , \end{aligned}$$

where $\alpha = \alpha (\vec {r})$ ($m^{-1}$) determines the strength of suppression, and ${\hat{S}} = {\hat{S}}(\vec {r}, t)$ indicates the estimated propagation direction. In order to motivate this modification, we first revert to the second order hyperbolic PDE by taking the temporal derivative of (4) and substituting Eq. (3):

5 $$\begin{aligned} \frac{\partial ^{2}{p}}{\partial {t}^{2}} = \rho c^2 \vec {\nabla }\cdot \left( \frac{1}{\rho } \vec {\nabla }p \right) - \alpha c \left( \frac{\partial {p}}{\partial {t}} + c {\hat{S}} \cdot \vec {\nabla }p \right) . \end{aligned}$$

Thus, we have obtained the acoustic wave equation with an extra $\alpha$-weighted, one-way term towards the ${\hat{S}}$ direction. This term corresponds to the term proposed by Fletcher et al.^5^, and has its origin in sponge-like boundary conditions^16^. This particular form was selected because it does not require any auxiliary fields.

To observe the effect of this extra term on the wave-field, we derive the solution of Eq. (5) for a plane wave traveling in the ${\hat{k}}$-direction:

6 $$\begin{aligned} P(\vec {k}, \omega ) = P_0(\vec {k}, \omega ) \exp \left[ i\left( \vec {k}\cdot \vec {r} - c\Vert \vec {k}\Vert t\right) \right] \exp \left[ -\frac{\alpha c t}{2} \left( 1 - {\hat{S}}\cdot {\hat{k}} \right) \right] , \end{aligned}$$

where $\vec {k}$ and $\omega$ denote the wave-vector and angular frequency of the plane-wave, respectively. A complete derivation of this result can be found in the supplementary information. In Eq. (6), we see that the proposed modification leads to a directional suppression effect. When ${\hat{S}}$ coincides with the plane wave’s direction, e.g: ${\hat{S}}\cdot {\hat{k}}=1$, the plane wave propagates unaltered. On the other hand, when ${\hat{S}}$ and ${\hat{k}}$ are opposite, e.g: ${\hat{S}}\cdot {\hat{k}}=-1$, the plane wave is maximally suppressed by a factor $\exp [-\alpha c t]$. In between these two extremes, suppression is proportional to the cosine of the angle between ${\hat{S}}$ and ${\hat{k}}$. Since this method solely suppresses reflections, and does not affect the transmitted amplitude, energy is not preserved. This method must thus be viewed as a non-physical acoustic wave equation.

The observations above motivate us to define a penetration depth, $\delta _p = \alpha ^{-1}$, for reflecting waves propagating in a direction exactly opposite to the incident direction. Alternatively, suppression can be viewed as a temporal process by defining a time-decay constant $\tau$, such that $\alpha (\vec {r}) = ( c(\vec {r}) \tau )^{-1}$.

## Finite difference implementation

The proposed method for reflection suppression is demonstrated using a ${\mathcal {O}}(t^2, x^4)$ staggered-grid FDTD implementation^17^ of Eqs. (3) and (4) with Perfectly Matched Layer (PML) boundary conditions^18^. The additional term in Eq. (4) is implemented using spatial (cubic) and temporal (quadratic) interpolation. The complete FD scheme can be found in the supplementary information.

The choice of FDTD provides the added benefit of allowing one to work with the acoustic Poynting vector, which can be used to determine the local wave-field propagation direction ${\hat{S}}(\vec {r}, t)$. Conveniently, the additional cost for computing and storing this quantity is low. In order to account for regions where the acoustic Poynting vector is ill-defined, we use the time-stacking technique described by Yoon et al.^7^.

Using the above method, the maximum value of $\alpha$ is defined based on the source frequency through a time constant $\tau$. Additionally, the value of $\alpha$ is set to 0 within a small circle around the source location, since the acoustic Poynting vector is not well-defined at the time of source-injection. In theory, $\tau$ can be kept constant throughout the medium. In practice, to minimize perturbations to the transmitted wave-field, it is recommended to scale $\tau$ with respect to the local medium velocity contrast, e.g: $\tau (\vec {r}) = \frac{max(|\vec {\nabla }c|)}{|\vec {\nabla }c|} \tau _{\text {min}}$, where $\tau _{\text {min}}$ denotes the fastest time-decay present in the medium.

## Results

**Figure 1:**
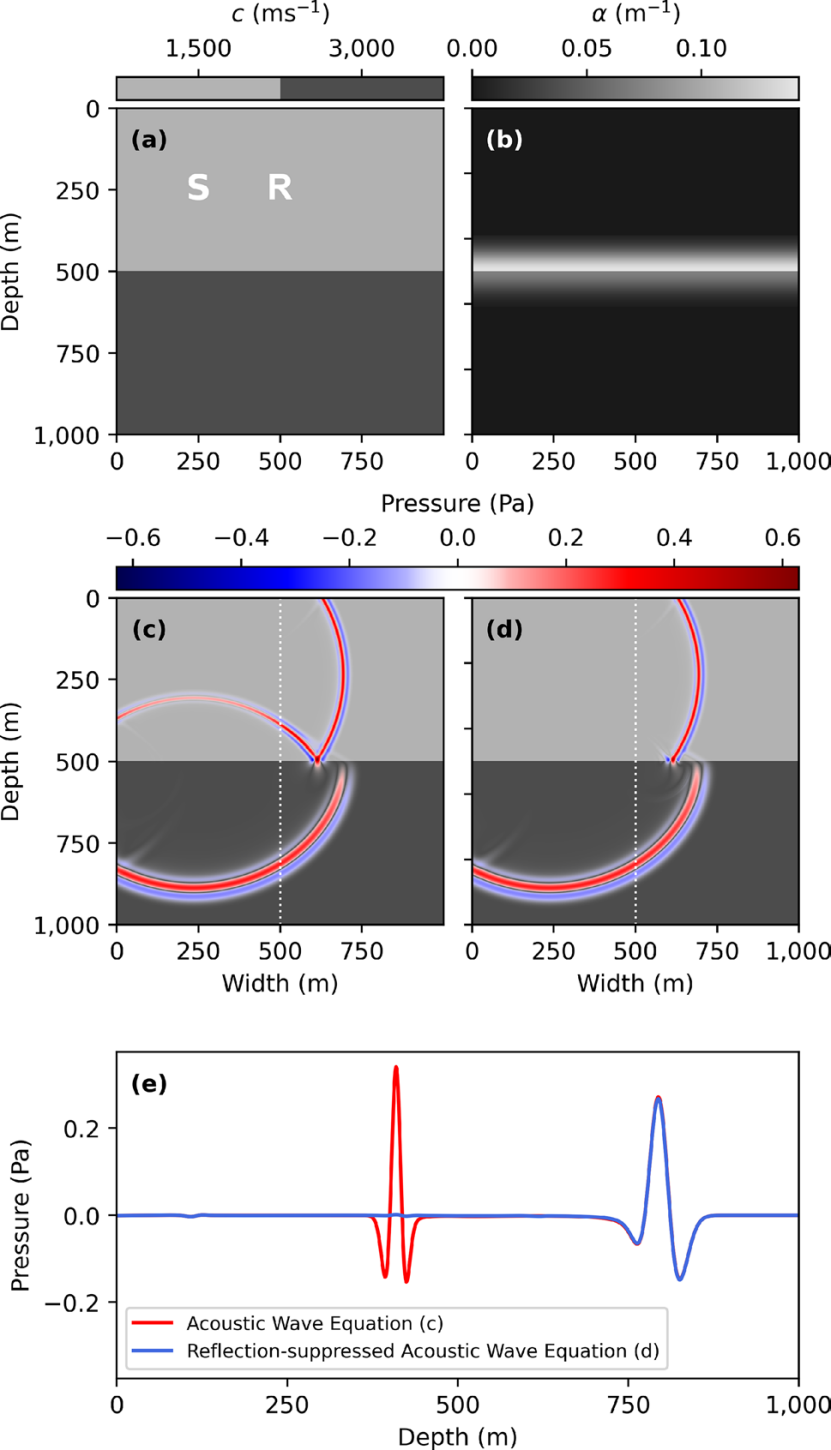
(**a**) The wave-speed of a 2 layer velocity-profile with point source location S and (**b**) local suppression constant $\alpha (\vec {r})$. (**c**) shows a snapshot of the wave-field at $t=0.35$ s for the acoustic wave Eqs. (1, 2) and (**d**) the reflection-suppressed wave Eqs. (3, 4), using a peak frequency of 50 Hz, 10 grid-points per smallest wavelength, and a CFL^20^ number of 0.5. The resulting space interval and step-size become $\Delta x = 1.0$ m and $\Delta t = 1.6 \cdot 10^{-4}$ s, respectively. (**e**) shows a cross-section through snapshots (**c**) and (**d**) along the white dotted-line. A video of all snapshots is available as supplementary material.

First, we examine a medium with a sharp velocity contrast at geophysical scale (Fig. 1a). A point-source Ricker^19^ wavelet with a peak frequency of 50 Hz is injected inside the medium, after which the wave-fields arising from Eqs. (1) and (2) are compared with their reflection-suppressed counterparts (3, 4), with a value of $\tau _{\text {min}}=4.68 \cdot 10^{-3} \ \text {s}$ such that $\alpha _{\text {max}}=0.14 \ \text {m}^{-1}$ (Fig. 1b).

In Fig. 1c,d,e we observe the effectiveness of the reflection suppression method via a snapshot and time series display. Reflections at both small and large incident angles are fully suppressed, while the refracted wave-field propagates with little to no perturbations.

The value of the suppression constant used in Fig. 1 was determined by a sensitivity assessment for the value of $\tau _{\text {min}}$.

**Figure 2:**
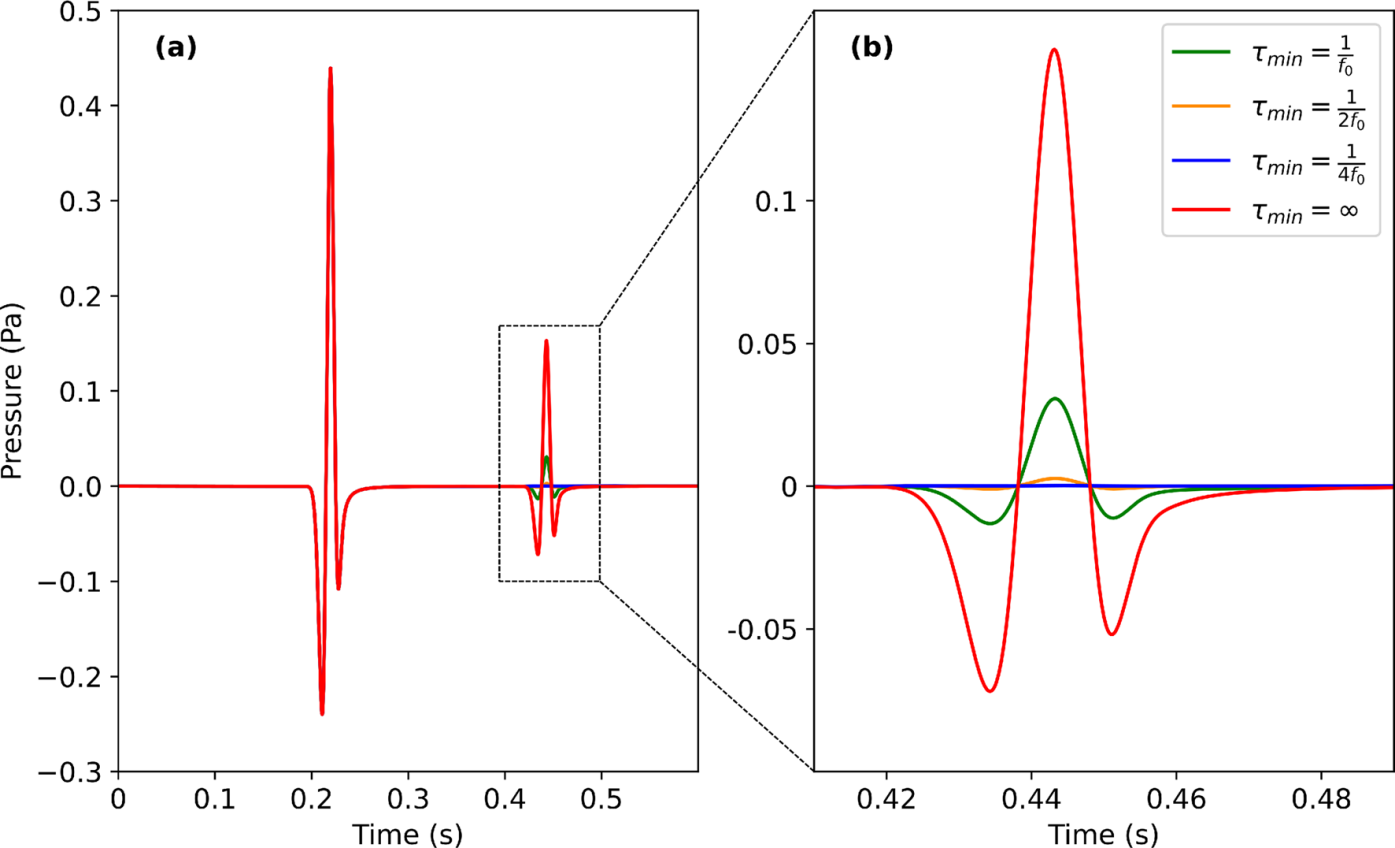
(**a**) The seismic trace, $p(\vec {r}_S, \vec {r}_R, t)$, obtained from the simulation of Fig. 1 from a point probe at location R (Fig. 1a) for different values of $\tau _{\text {min}}$. The first-arrival wave remains unaltered for all values of $\tau$, whereas the subsequent reflected wave from the interface depends strongly on $\tau$. Figure (**b**) highlights these suppressed reflected waves compared to the unmodified acoustic wave equation ($\tau _{\text {min}}=\infty$). The suppressed reflections yield an amplitude loss of − 14 dB for $\tau _{\text {min}}=\frac{1}{f_0}$, − 35 dB for $\tau _{\text {min}}=\frac{1}{2 f_0}$, and − 54 dB for $\tau _{\text {min}}=\frac{1}{4 f_0}$.

Figure 2 shows the level of suppression for different levels of $\tau _{\text {min}}$. Complete suppression of the reflections seen in Fig. 1a is reached at a value of $\tau _{\text {min}}=\frac{1}{4 f_0}$, or $\alpha _{\text {max}} = \frac{1}{4 c f_0}$. The level of suppression can be tuned depending on the level of velocity contrasts in the medium or in the case when there exist specific regions where reflections must be suppressed.

**Figure 3:**
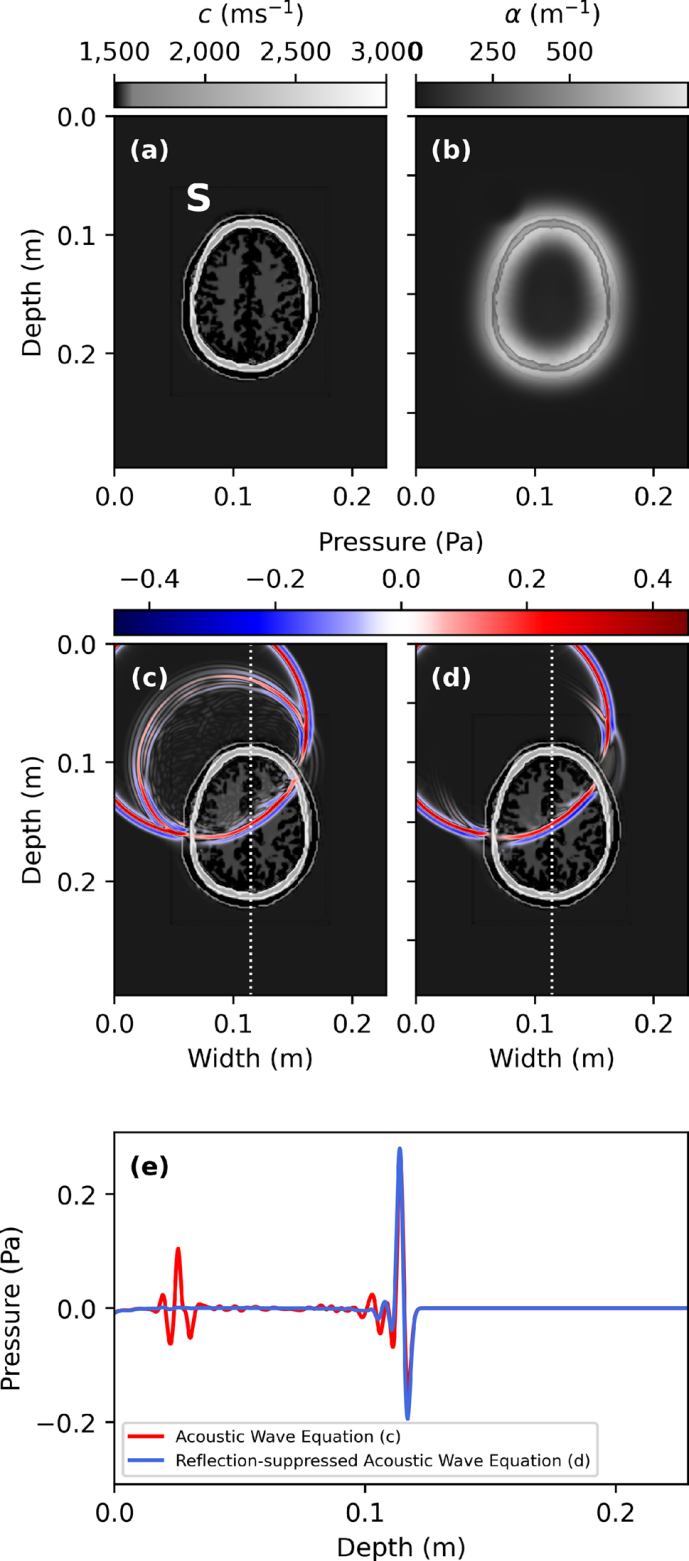
(**a**) The wave-speed of a human skull model with point source location S and (**b**) local suppression constant $\alpha (\vec {r})$. (**c**) shows a snapshot of the wave-field at $t=7.44 \cdot 10^{-5}$ s for the acoustic wave equation (1, 2) and (**d**) the reflection-suppressed wave equation (3, 4), using a peak frequency of 200 kHz, 10 grid-points per smallest wavelength, and a CFL^20^ number of 0.5. The resulting space interval and step-size become $\Delta x = 2.45 \cdot 10^{-4}$ m and $\Delta t = 5.32 \cdot 10^{-8}$ s, respectively. (**e**) shows a cross-section through snapshots (**c**) and (**d**) along the white dotted-line. A video of all snapshots is available as supplementary material.

Next, we apply the same methodology in the ultrasound regime to a human skull model^21^ (Fig. 3a) with a peak frequency of 200 kHz. We use a value of $\tau _{\text {min}}=7.80 \cdot 10^{-7} \ \text {s}$ such that $\alpha _{\text {max}}=855 \ \text {m}^{-1}$ (Fig. 3b).

In Fig. 3c,d,e we once again observe that the reflected wave-fields are very strongly suppressed, while the transmitted wave-fields only exhibit small perturbations with respect to the unmodified acoustic wave equation.

**Figure 4:**
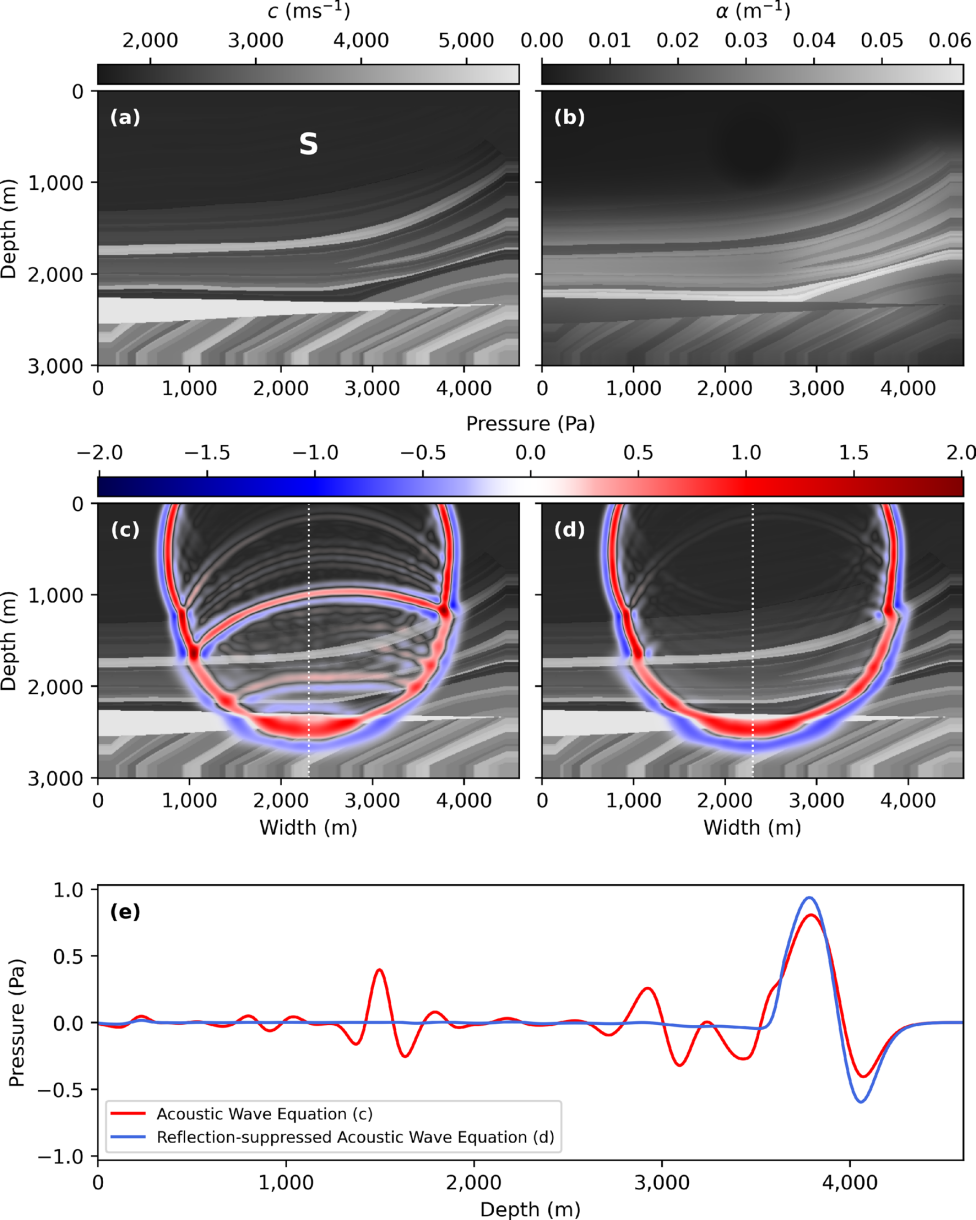
(**a**) The wave-speed of the Marmousi model with point source location S and (**b**) local suppression constant $\alpha (\vec {r})$. (**c**) shows a snapshot of the wave-field at $t=1.12$ s for the acoustic wave equation (1, 2) and (**d**) the reflection-suppressed wave Eqs. (3, 4), using a peak frequency of 10 Hz, 10 grid-points per smallest wavelength, and a CFL^20^ number of 0.5. The resulting space interval and step-size become $\Delta x = 5.70 \cdot 10^{-4}$ m and $\Delta t = 5.18 \cdot 10^{-4}$ s, respectively. (**e**) shows a cross-section through snapshots (**c**) and (**d**) along the white dotted-line. A video of all snapshots is available as supplementary material.

Lastly, we repeat the same procedure for the geophysical Marmousi^22^ model (Fig. 4a) at a peak frequency of 10 Hz. We use a value of $\tau _{\text {min}}=7.80 \cdot 10^{-3} \ \text {s}$ such that $\alpha _{\text {max}}=7.50 \cdot 10^{-2} \ \text {m}^{-1}$ (Fig. 4b).

We once again observe a complete suppression of reflected wave-fields. The transmitted wave-field of the modified acoustic wave equation in Fig. 4e contains varying perturbations with respect to the acoustic wave equation. In part, these perturbations can be explained due to the wave-front in Fig. 4c containing both reflected and transmitted wave-fields at heterogeneous locations, where Fig. 4d only contains the transmitted wave-field, making it difficult to compare the two figures.

## Discussion

The examples show that the method presented in this paper strongly suppresses internal reflections in a robust manner, even within highly heterogeneous media. In addition, the transmitted wave-fields exhibit only small perturbations. In order to fully remove these small perturbations, we recommend a combination of slowness smoothing and a contrast-dependent $\alpha$ to keep changes to the transmitted wave-field to a minimum. If desired, this method can also be used in conjunction with an impedance-matched wave equation, where the impedance is kept constant throughout the medium, e.g: $\rho (\vec {r})=c^{-1}(\vec {r})$. However, this will not allow for an independently chosen density contrast and significantly affects the transmission amplitudes. A comparison between our method and impedance matching is included in the supplementary information.

In general, the acoustic Poynting vector has shown to give an accurate estimate of the local propagation direction of wave-fields. However, problems may arise in the case of interfering waves. Firstly, we note that the use of the acoustic Poynting vector as a measure of wave-field direction breaks down for interfering waves. Secondly, and more importantly, we note that the modified Eq. (5) does not allow for reflection suppression in multiple directions simultaneously. For this reason, more sophisticated wave-field decomposition methods would not provide a solution to this issue. To remedy this, our method of contrast-dependent $\alpha$ allows interfering waves far away from areas exhibiting large contrasts in wave-speed to propagate unaltered. Furthermore, at high-contrast regions where interfering waves are known to appear, suppression could be turned off by setting $\alpha$ to zero locally.

As evident from the plane wave solution of Eqs. (3) and (4), it is possible for forward propagating components of the wave-field not exactly aligned with ${\hat{S}}$ to also be suppressed. The losses incurred from such misalignments, however, can be disregarded in general because of the exponential term in Eq. (6). Moreover, the results do not show any occurrence of suppression of the forward propagating wave-field.

As an alternative to the acoustic Poynting vector, a-priori ray-based methods such as Eikonal solvers can be used as a measure of the wave-field direction in the case of point-sources, by using the gradient of the shortest travel time as a time-independent propagation direction vector. It is important to note that in this way only primary arrivals are taken into account. Using this approach, the method presented here can also be applied in the frequency domain. After temporally Fourier transforming Eq. (5) we obtain a modified Helmholtz equation, which can subsequently be solved independently for each frequency component. Our experimental results using this approach show similar reflection suppression compared to the time domain method. Lastly, because PML’s in the space-frequency domain only require a small modification to the spatial gradient term, the suppressing term of Eq. (5) could conceivably also be implemented via PML’s inside the domain.

Computational costs for state-of-the-art FD wave simulations are of primary importance. The spatial interpolation step used in this method keeps the added computational cost to a minimum, by only using values which are already required to compute the derivatives of $v_x$ and $v_y$. Further improvements in computational speed can be achieved by using adaptive scheme approaches^23^. Alternatively, Eq. (5) can be directly implemented using a flux-limiter^24^ in a dimensional splitting approach. Results from both these methods are identical, but flux-limited schemes are significantly more expensive computationally, and thus are not preferred. The implementation of this method can also be extended naturally to the three dimensional case. Lastly, it is worth emphasising that this approach consists of an analytical modification to the acoustic wave equation. Therefore, approaches to numerical solutions are not limited to finite difference methods and could also be implemented using finite-element or finite-volume methods.

## Conclusion

The modification to the acoustic wave equation proposed in this paper successfully suppresses reflections within heterogeneous media, while the transmitted wave-field only exhibits small perturbations. Using a staggered grid FD scheme, the modified acoustic wave equation is implemented without significant additional computational cost. In combination with the acoustic Poynting vector, the wave-field is essentially dynamically guided through a reflection-less, heterogeneous medium. Although the solution is non-physical, this method is very suitable for use in RTM, where internal reflections often lead to imaging artifacts. Additionally, this method could serve as a tool for analysis purposes for many imaging methods, and could be used for any type of wave simulation application.

## Supplementary Information

Supplementary Information.

Supplementary Information.

Supplementary Information.

Supplementary Information.

Supplementary Information.

Supplementary Information.

Supplementary Information.
