# Supplementary material for: A non-reflecting wave equation through directional wave-field suppression and its finite difference implementation
Source: Sci Rep. 2022 Jan 10;12:407. doi: 10.1038/s41598-021-04064-3 (PMC8748939; doi:10.1038/s41598-021-04064-3)
Supplement: Supplementary file 1 — Supplementary Information. [file 41598_2021_4064_MOESM1_ESM.pdf]

Supplementary Information

In this supplementary information document, we outline the structure of the supplemented videos and offer a comparison with the impedance-matched wave equation method. Additionally, we derive the effect of the directional wave-field suppression method on a plane-wave, and discuss a finite difference staggered-grid implementation used to create the results of the main article.

Contents

1 Comparison with Impedance-matched Wave Equation **1**

2 Directional Wave-field suppression: Plane Wave Derivation **1**

3 Finite Difference Staggered Grid Implementation **2**

1 Comparison with Impedance-matched Wave Equation

The first 3 videos contain all snapshots from the models in the figures of the main article, labeled:

- `VIDEO_1_FIG1_2LAYER.mp4`
- `VIDEO_2_FIG3_SKULL.mp4`
- `VIDEO_3_FIG4_MARMOUSI.mp4`

In this supplementary document, we also compare the modified wave equation with the impedance-matched wave equation. Videos of the resulting snapshots can be found in the following 3 videos:

- `VIDEO_4_IMPMATCH_COMPARISON_2LAYER.mp4`
- `VIDEO_5_IMPMATCH_COMPARISON_SKULL.mp4`
- `VIDEO_6_IMPMATCH_COMPARISON_MARMOUSI.mp4`

From this, it can be seen that impedance-matching fails to suppress reflections at larger incident angles. Moreover, due to the local change in density, wave amplitudes are not preserved with respect to the original wave equation.

2 Directional Wave-field suppression: Plane Wave Derivation

In this section, we derive the effects of the directional wave-field suppression method for a plane wave. First, we define the temporal Fourier Transform:

$$\hat{P}(\omega,\vec{r}) = \int_{-\infty}^{\infty} dt\; p(t,\vec{r})e^{-i\omega t} \tag{1}$$

where ω is the temporal frequency in rad/s. Next, we consider our modified reflection-suppressed wave equation:

$$\frac{\partial^2 p}{\partial t^2} = \rho c^2 \vec{\nabla}\cdot\left(\frac{1}{\rho}\vec{\nabla}p\right) - \alpha\left(\rho c^2 \hat{S}\cdot\vec{\nabla}p + c\frac{\partial p}{\partial t}\right), \tag{2}$$

and take the temporal Fourier Transform to obtain a modified Helmholtz equation:

$$-\omega^2 P = \rho c^2 \vec{\nabla}\cdot\left(\frac{1}{\rho}\vec{\nabla}P\right) - \alpha\left(\rho c^2 \hat{S}\cdot\vec{\nabla}P - i\omega c P\right). \tag{3}$$

We now consider the dispersion relationship of our modified equation by inserting a plane wave solution $P(\vec{k},\omega) = P_0(\vec{k},\omega)\exp\left\{i\left(\vec{k}\cdot\vec{r}-\omega t\right)\right\}$ with angular frequency ω and wave-vector $\vec{k}$:

$$-\omega^2 = -c^2|k|^2 - i\alpha\left(-c\omega + c^2\hat{S}\cdot\vec{k}\right), \tag{4}$$

where $\vec{k} = (k_1, k_2, \ldots, k_n)$ are the spatial wave-numbers corresponding to the dimensions $\vec{r} = (r_1, r_2, \ldots, r_n)$. This equation can be solved exactly: (`https://www.wolframalpha.com/input/?i=solve+%28-omega%5E2%29+%3D+%28-+c%5E2*k%5E2+-++i*a*%28+-+c*omega+%2Bc%5E2*r%29%29+for+omega`)

$$\omega = -\frac{i\alpha c}{2} \pm \frac{c}{2}\sqrt{-\alpha^2 + 4i\alpha\hat{S}\cdot\vec{k} + 4|k|^2}. \tag{5}$$

Note for $\alpha = 0$, we obtain:

$$\omega = \pm c|k|, \tag{6}$$

which corresponds to dispersion relation for the unmodified wave equation.
Let us now introduce a "penetration depth" $\delta_p = \frac{1}{\alpha}$, $[\delta_p] = m$. We want to find what happens in the limit of weak suppression, e.g: $\frac{\delta_p}{c} = \frac{1}{\alpha c}$ becomes large. Rewriting $\gamma = \alpha c = \frac{c}{\delta_p}$ gives:

$$\omega = -\frac{i\gamma}{2} \pm \frac{1}{2}\sqrt{-\gamma^2 + 4ic\gamma\hat{S}\cdot\vec{k} + 4c^2|k|^2}. \tag{7}$$

A Taylor expansion of the square root around $\gamma = 0$ subsequently yields: (`https://www.wolframalpha.com/input/?i=taylor+expand+sqrt%28-g%5E2+%2B4*i*c*g*r+%2B+4*c%5E2*k%5E2%29+around+g%3D0`)

$$\sqrt{-\gamma^2 + 4ic\gamma\hat{S}\cdot\vec{k} + 4c^2|k|^2} \approx 2c|k| + i\gamma\hat{S}\cdot\hat{k}. \tag{8}$$

Returning to our expression for ω, for positive frequencies, we obtain:

$$\omega = c|k| - i\frac{\gamma}{2}\left(1 - \hat{S}\cdot\hat{k}\right). \tag{9}$$

Substituting this expression for ω in our plane wave solution $P(\vec{k},\omega) = P_0(\vec{k},\omega)\exp\left\{i\left(\vec{k}\cdot\vec{r} - \omega t\right)\right\}$ yields:

$$P(\vec{k},\omega) = P_0(\vec{k},\omega)\cdot\exp\left\{i\left(\vec{k}\cdot\vec{r} - c|k|t\right)\right\}\cdot\exp\left\{-\frac{\gamma t}{2}\left(1 - \hat{S}\cdot\hat{k}\right)\right\}. \tag{10}$$

Isolating the damping term and rewriting in terms of α gives:

$$\exp\left\{-\frac{\alpha ct}{2}\left(1 - \hat{S}\cdot\hat{k}\right)\right\}. \tag{11}$$

Therefore we find that we can also rewrite α in terms of a typical damping constant τ, $[\tau] = s$:

$$\alpha = \frac{1}{c\tau}, \tag{12}$$

such that a wave traveling in the opposite direction of $\hat{S}$ ($\hat{k} = -\hat{S}$) is damped by a factor $\exp\left\{-\frac{t}{\tau}\right\}$.

3 Finite Difference Staggered Grid Implementation

In this section, we briefly outline the staggered-grid, time-domain finite difference implementation of the modified wave equation of the main article. First, we consider the modified stress-strain relationship:

$$\begin{aligned} \frac{\partial\vec{v}}{\partial t} &= -\frac{1}{\rho}\vec{\nabla}p \\ \frac{\partial p}{\partial t} &= -\rho c^2\vec{\nabla}\cdot\vec{v} - \alpha\left(cp - \rho c^2\vec{\hat{S}}\cdot\vec{v}\right). \end{aligned} \tag{13}$$

While the first equation can easily be discretized on a staggered grid, the second is more difficult. To find a proper discretization, the following scheme for the second equation is proposed.

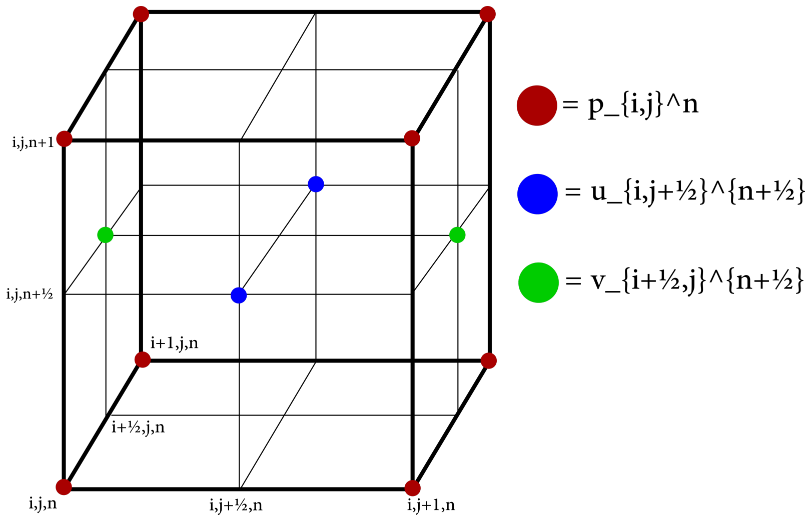

Figure 1. Awakawa C-grid

We use a half-stepping procedure, where we define p on full grid-points and full time-steps: $p_{i,j}^{n}$, and $\vec{v}$ on half grid-points and half time-steps: $u_{i,j+\frac{1}{2}}^{n+\frac{1}{2}}$ and $v_{i+\frac{1}{2},j}^{n+\frac{1}{2}}$. In this way, the stepping procedure can make use of centered differences. Writing the second equation at time index $n+\frac{1}{2}$ yields:

$$\left[\frac{\partial p}{\partial t}\right]_{i,j}^{n+\frac{1}{2}} = -\rho_{i,j}(c_{i,j})^2\left[\frac{\partial u}{\partial x}+\frac{\partial v}{\partial z}\right]_{i,j}^{n+\frac{1}{2}} - \alpha_{i,j}c_{i,j}\left(p_{i,j}^{n+\frac{1}{2}} - \rho_{i,j}(c_{i,j})^2\left(S_{x_{i,j}}\vec{u}_{i,j}^{n+\frac{1}{2}} + S_{z_{i,j}}\vec{v}_{i,j}^{n+\frac{1}{2}}\right)\right). \tag{14}$$

We approximate the temporal derivative to second order using centered differences:

$$\left[\frac{\partial p}{\partial t}\right]_{i,j}^{n+\frac{1}{2}} = \frac{1}{\Delta t}\left[p_{i,j}^{n+1} - p_{i,j}^{n}\right]. \tag{15}$$

Additionally, we approximate the spatial derivatives to fourth order using centered differences:

$$\begin{aligned}\left[\frac{\partial u}{\partial x}\right]_{i,j}^{n+\frac{1}{2}} &= \frac{1}{\Delta x}\left[\frac{1}{24}u_{i,j-\frac{3}{2}}^{n+\frac{1}{2}} - \frac{9}{8}u_{i,j-\frac{1}{2}}^{n+\frac{1}{2}} + \frac{9}{8}u_{i,j+\frac{1}{2}}^{n+\frac{1}{2}} - \frac{1}{24}u_{i,j+\frac{3}{2}}^{n+\frac{1}{2}}\right]\\ \left[\frac{\partial v}{\partial z}\right]_{i,j}^{n+\frac{1}{2}} &= \frac{1}{\Delta x}\left[\frac{1}{24}v_{i-\frac{3}{2},j}^{n+\frac{1}{2}} - \frac{9}{8}v_{i-\frac{1}{2},j}^{n+\frac{1}{2}} + \frac{9}{8}v_{i+\frac{1}{2},j}^{n+\frac{1}{2}} - \frac{1}{24}v_{i+\frac{3}{2},j}^{n+\frac{1}{2}}\right].\end{aligned} \tag{16}$$

Subsequently, we spatially interpolate u and v using cubic interpolation:

$$\begin{aligned}u_{i,j}^{n+\frac{1}{2}} &= -0.0625u_{i,j-\frac{3}{2}}^{n+\frac{1}{2}} + 0.5625u_{i,j-\frac{1}{2}}^{n+\frac{1}{2}} + 0.5625u_{i,j+\frac{1}{2}}^{n+\frac{1}{2}} - 0.0625u_{i,j+\frac{3}{2}}^{n+\frac{1}{2}}\\ v_{i,j}^{n+\frac{1}{2}} &= -0.0625v_{i-\frac{3}{2},j}^{n+\frac{1}{2}} + 0.5625v_{i-\frac{1}{2},j}^{n+\frac{1}{2}} + 0.5625v_{i+\frac{1}{2},j}^{n+\frac{1}{2}} - 0.0625v_{i+\frac{3}{2},j}^{n+\frac{1}{2}}\\ &.\end{aligned} \tag{17}$$

Lastly, we temporally interpolate p using quadratic interpolation:

$$p_{i,j}^{n+\frac{1}{2}} = -0.125p_{i,j}^{n-1} + 0.75p_{i,j}^{n} + 0.375p_{i,j}^{n+1}. \tag{18}$$

Putting it all together leads to an expression for the next time step $p_{i,j}^{n+1}$.
